# Genetic liability to major psychiatric disorders contributes to multi-faceted quality of life outcomes in children and adults

**DOI:** 10.1038/s41398-025-03443-y

**Published:** 2025-07-07

**Authors:** Yingjie Shi, Nina Roth Mota, Barbara Franke, Emma Sprooten

**Affiliations:** 1 Department of Human Genetics, Radboud University Medical Center, Nijmegen, The Netherlands; 2 Donders Institute for Brain, Cognition and Behaviour, Radboud University, Nijmegen, The Netherlands; 3 Department of Medical Neuroscience, Radboud University Medical Center, Nijmegen, The Netherlands

**Keywords:** Genomics, Psychiatric disorders

## Abstract

Psychiatric conditions, known for their hereditary nature, exert significant impacts on various life domains. Leveraging this heritability, we examine the relations between genetic susceptibility to major psychiatric disorders and the multifaceted aspects of quality of life in two population-based cohorts, the Adolescent Brain Cognitive Development (ABCD) study (N = 3909 preadolescent children) and the UK Biobank (N = 269,293 adults). Genetic susceptibility to seven major psychiatric disorders was quantified by polygenic scores derived from extensive genome-wide association studies (N = 21,000–413,000). Pervasive associations were found between genetic risk for all seven major psychiatric disorders investigated and age-relevant real-life quality of life indices, with varied patterns of associations for different life domains. We especially highlight the role of genetic risks for ADHD and major depressive disorders. Our findings emphasize the continuous nature of psychiatric traits, extending their influence on daily life experiences and societal functioning beyond symptomatology and diagnostic classifications.

## Introduction

The impact of psychiatric disorders transcends the confines of mental wellbeing, exerting influence across a multifaceted spectrum of life domains, encompassing educational, occupational, physical, social, and psychological outcomes [1]. This comprehensive reach is well-documented in extensive epidemiological studies, which consistently associate psychiatric disorders with a decrement in overall quality of life and specific functional impairments [2, 3]. Crucially, these burdens extend not only to individuals who meet the clinical symptom and impairment thresholds for a psychiatric diagnosis but also to those who exhibit subclinical symptomatology. This phenomenon aligns with the liability threshold model in psychiatric genetics [4], which posits that the genetic risk for psychiatric disorders exists on a continuum, and individuals may experience varying degrees of genetic susceptibility, irrespective of clinical diagnosis.

In the pursuit of enhancing citizens’ well-being and satisfaction, many nations have made quality of life improvement a central objective of their policy agendas. However, there exists no universally accepted framework for conceptualizing and measuring quality of life. A commonly endorsed model recognizes quality of life as a construct encompassing dimensions of standard of living that conform to societal expectations. This is quantifiable across domains such as social, health, and economic well-being, in addition to subjective assessments of personal well-being, reflecting the degree to which individual life expectations are met [5]. Quality of life, however, is not a static construct and can vary significantly across different life stages, particularly between childhood and adulthood. In children, physical health and safety are paramount, as are education and social interactions that foster cognitive and emotional development [6, 7]. These elements are critical in shaping a child’s foundational sense of well-being and future prospects. In adulthood, economic stability, career satisfaction, and the quality of personal relationships become more important indicators of quality of life [8]. In addition to these stage-specific factors, core elements like health and social connections remain consistently important throughout the lifespan [1, 6], highlighting both the dynamic and enduring nature of quality of life.

Large-scale genome-wide association studies (GWASs) have enabled the identification of common genetic variation contributing to psychiatric disorders [9]. The polygenic architecture of many prevalent and debilitating psychiatric disorders has been characterized with large GWAS samples, including attention-deficit/hyperactivity disorder (ADHD) [10], autism spectrum disorder (ASD) [11], major depressive disorder (MDD) [12], anxiety disorders (ANX) [13, 14], schizophrenia (SCZ) [15], bipolar disorder (BIP) [16], and cannabis use disorder (CUD) [17]. Combining information across the genome through a weighted sum of the number of the disorder-associated alleles, polygenic scores (PGSs) [18] can provide proxies for individuals’ genetic loading for a psychiatric condition on a continuous scale in the population. Such quantification of genetic susceptibility to different phenotypes has proven useful in risk stratification of common complex diseases [19], and is increasingly powerful as GWAS sample sizes increase. Prior research [20] demonstrated that genetic predisposition to schizophrenia contributes to additional variance in quality of life beyond clinical factors. However, there is still a need for systematic quantification of PGS effects across psychiatric conditions on diverse quality of life domains.

Here, we aimed to assess how genetic susceptibility to seven major psychiatric disorders indexed by PGSs relates to various quality of life-relevant indices at different phases of the lifespan, specifically preadolescent children and middle-aged adults. To this end, we took advantage of two large population cohorts, the Adolescent Brain Cognitive Development (ABCD) study and the UK Biobank, and derived multi-faceted quality of life constructs at these two life stages, capturing general and specific domains of human functioning and experiences.

## Methods

### Participants

Our study sample consisted of 3909 non-Hispanic white preadolescent children (47% females, age 9.92 ± 0.62 years) recruited across the United States of America as part of the ABCD study cohort (request 11315, data release 4.0) and 269,293 white British, unrelated adults (54% females, age 56.95 ± 7.94 years) from the population-based UK Biobank (application 23668, data release 3.0). Participants and their caregivers in the studies provided written or verbal consent, as appropriate.

### Quality of life outcomes

We hypothesized several models capturing different quality of life domains in both cohorts, and assessed their fit through confirmatory factor analyses. Given the great phenotyping depth of both cohorts, more than one well-fitting model structure with sets of reasonable indicators was available. Here, for each cohort, we selected one theoretically sound and statistically best-fit model as the primary model used in the main analyses, as described in the main text, and present a less well-fitting alternative model in eFig. 1.

In the ABCD cohort, we fitted the model with nine observed variables (eTable 1) indicating three latent factors – educational performance and cognition (Edu), physical health (Hea1), and adverse peer experience (Peer). Observed variables are, for Edu, school grades in the past year reported by parents (sag_grade_type) and youths (sag_grades_last_yr), cognition total composite score (abcd_tbss01:nihtbx_totalcomp_uncorrected); for Hea1, ever seen doctors before the past year (excluding regular check-ups) (abcd_mx01:medhx_1b), ever seen doctor for any (severe) diseases (derived from abcd_mx01), emergency room visits before the past year (derived from abcd_mx01), ever been in the hospital overnight or longer (abcd_mx01:medhx_8a); for Peer, ever been cyberbullied (abcd_cb01:cybb_phenx_harm), experienced victimization from peers (derived from abcd_peq01). We used variables from the second-year follow-up, except for medical history reports and cognition total composite score, which were only available as baseline measures and expected to capture the underlying traits that are relatively stable across time. Individuals who reported ‘do not know’, ‘not applicable’, or ‘refuse to answer’ to the abovementioned items were excluded from the analyses. A final list of 3909 participants with phenotypic data available was included in downstream analyses.

In the UK Biobank cohort, we included three latent factors – socioeconomic status (SES), health (Hea2), and social wellbeing (Soc) – which were indicated by eight binary or ordinal variables (eTable 2): average total household income before tax (UK Biobank index 738) and educational qualifications (6138) for the first factor; overall health rating (2178), long-standing illness, disability or infirmity (2188), and whether diagnosed with any serious medical conditions (derived by aggregating item 6150, 6152, 2443, 2453, and 2473) for the second factor; the frequency of being able to confide with someone (2110), whether often feel loneliness or in isolation (2020), and the tendency to worry too long after embarrassment (1930) for the third factor. For educational qualifications, we converted each individual’s highest qualification to an International Standard Classification of Education (ISCED) category and removed other qualifications that were not included in such classification scheme. Individuals who reported ‘do not know’ or ‘prefer not to answer’ to the abovementioned items were excluded from the analyses.

### Confirmatory factor analysis

The CFA models were implemented in lavaan package [21] using the method of weighted least squares mean and variance adjusted (WLSMV), which used diagonally weighted least squares to estimate model parameters, but the full weight matrix to compute robust standard errors. Final models contained the abovementioned three first-level factors and one second-level general QoL factor. To ease the interpretation, variables were sign-flipped so that larger values always correspond to higher quality of life levels. The variances of all latent variables (i.e., ‘factor scores’) in the models were fixed to unity, and their estimated values were computed using Empirical Bayes Method (EBM) [21]. The metrics of Comparative Fit Index (CFI), Root Mean Square Error of Approximation (RMSEA) and 90% confidence intervals, Standardized Root Mean Square Residual (SRMR), together with Tucker-Lewis Index (TLI) were employed to assess the model fit. CFI values above 0.95, RMSEA values below 0.06, SRMR values below 0.08, and TLI values above 0.95 were considered as evidence for good model fit [22].

### GWAS data

In selecting phenotypes for our study, we employed the following criteria: diversity in ages of onset, variety in symptom profiles, robust availability of large and recent GWAS datasets of European ancestry, and the ability to avoid sample overlap with our target cohorts. Guided by these considerations, we included seven major psychiatric disorders—ADHD, ASD, MDD, ANX, SCZ, BIP, and CUD—and ensured that all GWAS summary statistics excluded UK Biobank participants when computing PGSs for the UK Biobank. Notably, we chose CUD to represent substance use disorders because of its substantial sample size (N ≈ 358,000) and strong genetic correlations with other SUDs (e.g., r_g_ = 0.75 with alcohol use disorder, r_g_ = 0.74 with opioid use disorder, and r_g_ = 0.63 with tobacco use disorder) [23]. All GWAS summary statistics were based on cohorts of European ancestry, and were annotated to the Genome Reference Consortium (GRCh) 37/hg19 build. We reviewed the study protocols for each GWAS analysis to ensure good quality of summary statistics and sufficient SNP heritability of traits (h^2^_SNP_ > 0.05) before including them in the current study. An overview of the discovery GWASs with publication references is provided in Table 1. We removed strand-ambiguous SNPs, duplicated SNPs, multi-allelic SNPs, and SNPs with out-of-bounds values, or with MAF < 0.01, imputation quality INFO < 0.9, or not matching to HapMap3 reference panel.

**Table 1:** Overview of base GWAS summary statistics used in ABCD and UK Biobank cohorts.

GWAS phenotype	Publication	#cases		#controls		h^2^_SNP_ (SE)	Population prevalence
		ABCD as target sample	UK Biobank as target sample	ABCD as target sample	UK Biobank as target sample		
ADHD	Demontis et al., Nature Genetics (2023)	38,691		186,843		0.140 (0.010)	0.050
ASD	Matoba et al., Translational Psychiatry (2020)	18,382		27,969		0.118 (0.010)	0.012
MDD	Wray et al., Nature Genetics (2018)	59,851	45,591	113,154	97,674	0.087 (0.004)	0.150
ANX	Purves et al., Molecular Psychiatry (2020), Otowa et al., Molecular Psychiatry (2016)	31,977	7016	82,114	14,745	0.133 (0.011)	0.200
SCZ	Trubetskoy et al., Nature (2022)	53,386		77,258		0.240 (0.007)	0.010
BIP	Mullins et al., Nature Genetics (2021)	41,917	40,463	371,549	313,436	0.186 (0.008)	0.020
CUD	Johnson et al., Lancet Psychiatry (2020)	14,080		343,726		0.067–0.121 (0.006–0.011)	0.048

For MDD and BIP, UK Biobank samples were also included in the original GWAS analyses. To avoid bias introduced by sample overlap between base and target cohorts, we used summary statistics provided by the authors after removing UK Biobank participants. For ANX, summary statistics without UK Biobank cohort were provided by Otowa et al., Molecular Psychiatry (2016).

*h*^*2*^_*SNP*_ SNP heritability, *SE* standard error.

### Polygenic score calculation and statistical analyses

Detailed descriptions of the QC and imputation steps in both cohorts have been provided in previous publications [24, 25]. Subsequent sample and variant filtering was conducted prior to polygenic score analyses based on the protocol described in eMethods. We adopted PRS-CS [26] as the primary approach to compute polygenic scores for the four quality of life factors. PRS-CS used a Bayesian continuous shrinkage method to adjust SNP effect estimates from the original GWAS summary statistics and infer posterior SNP weights. We then used the --score command in PLINK 2.0 to sum over all SNPs weighted by their posterior effect sizes and derive an additive score per individual. Two other PGS approaches were also tested to further validate the results (eMethods), and support power analysis as described in the following section.

Simple linear regression was performed between each polygenic score (as predictor) and quality of life factor (as outcome). Sex, age in years, batch, site, and the first ten ancestry informative genotype PCs for the UK Biobank cohort and batch, site, plate, sex, age in months, and the first ten ancestry informative genotype PCs for the ABCD cohort were included as covariates. R^2^ was calculated for each PGS by subtracting variance explained by the covariates from variance explained by the full model including both the PGS and covariates. Multiple regression models with all 7 PGSs were further constructed to assess the overall variance explained by the different PGSs altogether on top of the covariates. Bonferroni correction was applied accounting for the number of polygenic scores and outcome variables tested. To assess the effect of differential sample sizes of the base GWAS samples, we performed power calculation for the PGS analyses as described in eMethods.

### SNP heritability estimation and genetic correlation analyses

Genome-wide association analyses on the four estimated latent factors were conducted in the UK Biobank cohort using PLINK 2.0 [27], with an assumed additive genetic model. Generalized linear regression model was performed on the imputed data, with sex, age, batch, plate, site, and the first ten PCs as the covariates. SNP-heritability estimates of each latent factor and their genetic correlations with the seven psychiatric disorders were then calculated using linkage disequilibrium score regression (LDSC) v1.0.1 [28] and LD scores precomputed from the European reference samples from the 1000 Genomes Project.

## Results

**Fig. 1:**
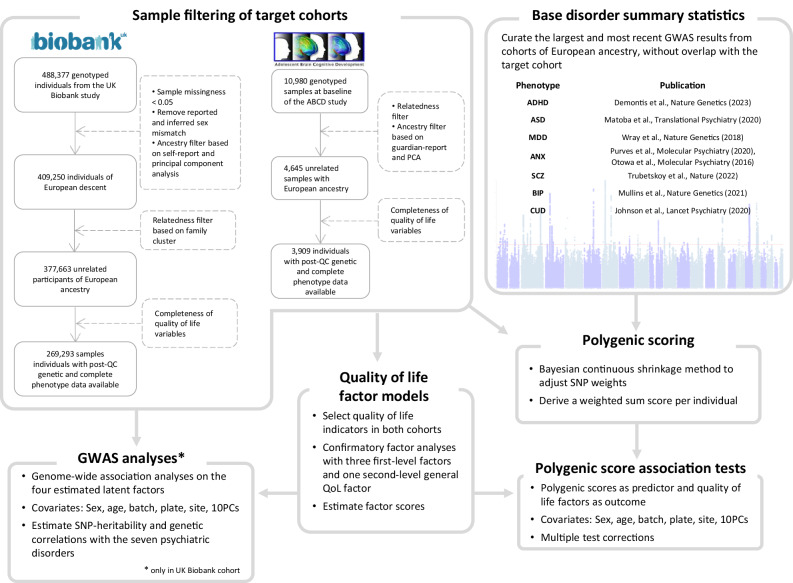
Sample curation and data analysis flow chart. The key analytic steps performed in the study were described in a modular fashion, with the arrows illustrating the input and output data involved in different steps. Dashed arrows and boxes denote filters applied to obtain the final analytic sample. QC quality control, PCA principal component analysis, PCs principal components, ADHD attention-deficit/hyperactivity disorder, ASD autism spectrum disorder, MDD major depressive disorder, ANX anxiety disorder, SCZ schizophrenia, BIP bipolar disorder, CUD cannabis use disorder.

**Fig. 2:**
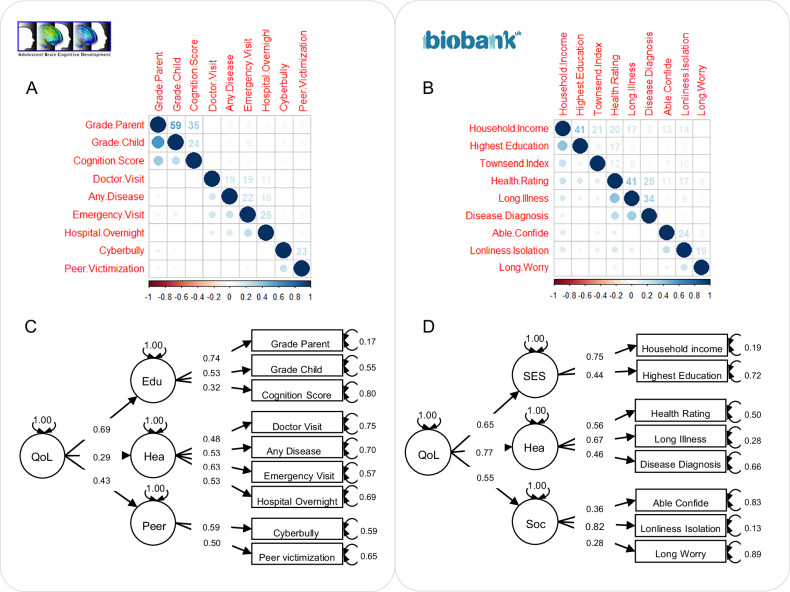
Quality of life factor structure. Correlation matrices of quality of life indicators included in either primary or alternative models for ABCD (**A**) and UK Biobank (**B**) cohorts. Primary confirmatory factor analysis model structures for ABCD (**C**) and UK Biobank (**D**) cohorts. Observed variables (indicators) are represented in rectangular boxes. Unobserved (latent) variables derived from the models are shown in circles. Single-headed arrows denote the influence of latent variables on indicators (or another latent variable), with the values on the arrows represent factor loading coefficients. The double-headed arrow connecting the variable to itself denotes residual variances. Higher scores of the indicators correspond to higher levels of quality of life when constructing the models. A pair of alternative models is presented in eFigure 1. QoL quality of life, Edu educational performance and cognition, Hea1 physical health modeled in ABCD cohort, Peer peer experience, SES socioeconomic status, Hea2 physical health modeled in UK biobank cohort, Soc social wellbeing.

The main data processing and analytic processes were illustrated in Fig. 1. In a final sample of 3909 preadolescent children and 269,293 adults for which both genotype data and quality of life factor scores were available, we modeled the covariances of the quality of life indicators (correlation matrices shown in Fig. 2A and Fig. 2B) and assessed the fit of the hypothesized model structures using confirmatory factor analysis (CFA). In the ABCD cohort, the model consisted of three first-order latent factors, namely educational performance and cognition (Edu), physical health (Hea1), and peer experience (Peer), as well as one second-order general quality of life (QoL) factor. This model had an excellent model fit (CFI = 0.989, RMSEA = 0.021, SRMR = 0.030, TLI = 0.984) (Fig. 2C). A similarly structured second-order model was estimated in the UK Biobank cohort (CFI = 0.971, RMSEA = 0.046, SRMR = 0.043, TLI = 0.952), with socioeconomic status (SES), physical health (Hea2), and social wellbeing (Soc) factors on the first-order level and general QoL factor on the second-order level (Fig. 2D). All model parameters are presented in eTable 3 and eTable 4. In a subset of the UK Biobank sample, in which self-rated life satisfaction measures were available, the three first-level quality of life factors estimated from the model structure were significantly associated with subjective satisfaction in their corresponding life domain (i.e., SES with individuals’ financial situation satisfaction, ρ = 0.25, p < 0.001; physical health with health satisfaction, ρ = 0.58, p < 0.001; social wellbeing with general happiness in life, ρ = 0.41, p < 0.001, family relationship, ρ = 0.32, p < 0.001, and friendship satisfaction, ρ = 0.28, p < 0.001) (eTable 5).

**Fig. 3:**
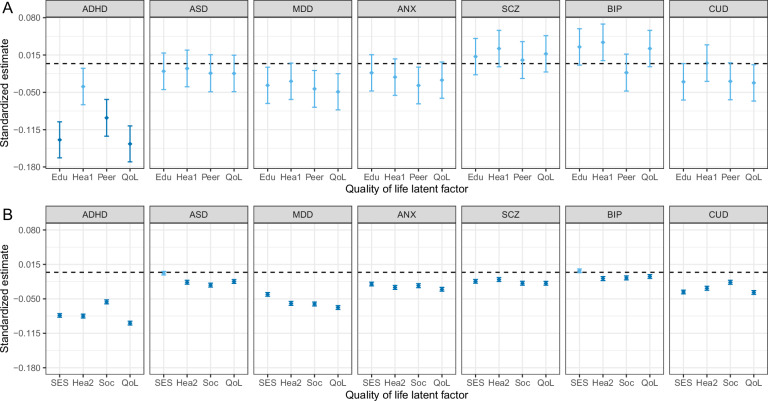
Associations between polygenic scores and quality of life latent factors. Regression estimates between polygenic scores for different major psychiatric disorders and quality of life latent factors for ABCD study **(A)** and UK Biobank **(B)** cohorts. ADHD attention-deficit/hyperactivity disorder, ASD autism spectrum disorder, MDD major depressive disorder, ANX anxiety disorder, SCZ schizophrenia, BIP bipolar disorder, CUD cannabis use disorder, QoL quality of life, Edu educational performance and cognition, Hea1 physical health modeled in ABCD cohort, Peer peer experience, SES socioeconomic status, Hea2 physical health modeled in UK Biobank cohort; Soc social wellbeing. Dark blue indicates statistical significance after correction. Error bars represent standard errors.

We examined patterns of associations between PGSs for psychiatric disorders and four latent quality of life factors estimated from the fitted model. In the ABCD cohort, the PGS for ADHD significantly explained 1.67% of Edu (β = −0.133, SE = 0.016, p = 1.53 × 10^−16^), 0.84% of Peer (β = −0.094, SE = 0.016, p = 7.81 × 10^−9^), and 1.85% of general QoL factors (β = −0.140, SE = 0.016, p = 3.37 × 10^−18^), respectively. The PGSs for other disorders were not significantly associated with any of the latent factors (Fig. 3A, eTable 6). In the UK Biobank cohort, PGSs based on all seven psychiatric disorders were associated with the general QoL factor and at least one first-order subdomain (Fig. 3B, eTable 6). Among them, ADHD-PGS showed the largest effect on the general QoL factor (β = −0.096, SE = 0.002, p < 2.23 × 10^−308^, R^2^ = 0.009), Hea2 (β = −0.083, SE = 0.002, p < 2.23 × 10^−308^, R^2^ = 0.007), and SES (β = −0.081, SE = 0.002, p < 2.23 × 10^−308^, R^2^ = 0.007). The largest effect on the Soc subdomain was seen with the MDD-PGS (β = −0.060, SE = 0.002, p = 9.96 × 10^−214^, R^2^ = 0.004), which was also associated with the domains of Hea2 (β = −0.059, SE = 0.002, p = 4.48 × 10^−211^, R^2^ = 0.003) and SES (β = −0.042, SE = 0.002, p = 3.63 × 10^−125^, R^2^ = 0.002). Genetic risk for CUD was most strongly associated with SES (β = −0.037, SE = 0.002, p = 2.05 × 10^−99^, R^2^ = 0.001), compared to the other two subdomains, whereas genetic risk for ASD was linked most strongly to the Soc subdomain (β = −0.024, SE = 0.002, p = 2.06 × 10^−36^, R^2^ = 0.001), and ANX to Hea2 (β = −0.028, SE = 0.002, p = 4.57 × 10^−51^, R^2^ = 0.001).

To evaluate the influence of statistical power (including differences in discovery GWAS sample size) on identifying PGS effects, the lower bound of the power estimates was obtained in both cohorts based on the sample sizes of both the base and target cohorts, SNP heritability, and trait prevalence. The power estimates in the UK Biobank cohort for all disorders and target phenotypes were above 0.990 across genetic correlation (r_g_) thresholds of 0.2, 0.4, 0.6, 0.8, and 1. In the ABCD cohort, all disorders except ASD and CUD were sufficiently powered for r_g_ = 0.4 and above. Assuming the same degree of genetic correlations with quality of life domains across disorders, the ranking of the seven disorders based on their power estimates would be as follows: ANX, SCZ, BIP, ADHD, MDD, CUD, and ASD. Full results of the power analyses are shown in eTables 7 and eTable 8. Further, the results were consistent across three polygenic scoring methods examined (eFigure 2 and eFigure 3).

Using the estimated latent quality of life factors as the target phenotype in the UK Biobank cohort, we further conducted GWAS and estimated the SNP heritability (*h*^*2*^_SNP_) of the latent factors using LD score regression. The estimated *h*^*2*^_SNP_ of the latent factors are in line with estimates for the individual indicators (SES: h^2^_SNP_ = 0.113 (0.005), Hea2: h^2^_SNP_ = 0.094 (0.004), Soc: h^2^_SNP_ = 0.063 (0.003), QoL: h^2^_SNP_ = 0.115 (0.005)). In the multiple regression model, seven PGSs together explained R^2^ = 0.011, 0.010, 0.006, 0.014 variances, which is 9.7%, 10.4%, 9.7%, and 12.2% of the SNP heritability of SES, Hea2, Soc, and general QoL, respectively. Variances explained proportional to SNP heritability by single PGSs are presented in eTable 9. To explore the genetic relationships between psychiatric disorders and quality of life domains, we estimated their pairwise genetic correlations. Consistent with PGS analysis results, a widespread pattern of negative associations was observed across quality of life domains (eTable 10). MDD (largest r_g_ = −0.688 for Soc factor), ANX (largest r_g_ = −0.682 for Soc factor), and ADHD (largest r_g_ = −0.633 for general QoL factor) were highly negatively genetically correlated with all the quality of life factors. CUD was most strongly correlated with SES (r_g_ = −0.400), and ASD with Soc (r_g_ = −0.298). The genetic correlations for SCZ and BIP were weaker, with BIP exclusively associated with the Hea2 subdomain (r_g_ = −0.133); no significant genetic correlation was seen between ASD and SES.

## Discussion

In this study, we set out to systematically evaluate the impact of psychiatric genetic liabilities on the quality of life in individuals during two distinct phases of life. We utilized PGSs that encompassed seven major psychiatric disorders and characterized their relations with various aspects of quality of life, with a specific focus on domains of academic performance, socio-economic factors, physical health, and social wellbeing. Our results unveiled a pervasive yet varied pattern of associations with quality of life across different psychiatric disorders, such that ADHD was more strongly associated with educational performance and cognition in children and with socioeconomic status in adults; while MDD was most strongly associated with social well-being in adults only. Notably, we accentuated the prominent role played by genetic burden for ADHD during childhood, while also highlighting the impact of genetic predisposition associated with ADHD, MDD, and CUD in adulthood.

Our study revealed that the impact of genetic susceptibility, as measured by PGSs, on quality of life exists across the lifespan, even in individuals without psychiatric diagnoses within the general population. This aligns with the observations of attenuated impairments in unaffected family members in twin or family studies. For instance, lower cognitive functioning has been observed in unaffected first-degree relatives of patients with schizophrenia [29], bipolar disorder [30], and ADHD [31], compared to healthy controls. While there is little research, and no evidence in identifying reductions in quality of life-related traits in high-risk family studies [32], our results revealed the overarching trend of decreased everyday functioning relating to genetic risks across disorders, encouraging further efforts exploiting the continuous nature of psychiatric traits.

To our knowledge, this is the first study to quantify the contribution of genetic risks across major psychiatric disorders to different aspects of quality of life. The quality of life domains we found associated with the genetic risk for each disorder were consistent with previous findings from case-control group contrasts based on clinical diagnosis [33–36]. For instance, a meta-analysis of over 6000 youths and their parents found that children with ADHD were most strongly impacted in aspects of academic performance and social functioning. Their physical functioning was only slightly lower, but the discrepancy compared to typically developing peers became larger as they grew older [36], consistent with our finding that higher genetic liability for ADHD is more strongly associated with compromised health status in adults than in children, whereas the academic and social domains were impacted both in children and adults. Notably, the lack of associations for other disorders in the pre-adolescent children’s cohort is likely not fully explained by statistical power, highlighting a potential temporal distinction in the manifestation of genetic risks for different psychiatric disorders. ADHD genetic risk is more likely to manifest early in life, significantly influencing brain development processes such as cortical maturation, particularly in the prefrontal cortex—which is crucial for executive functions including attention and motor planning [37]. Genetic risks for disorders such as MDD and SCZ may be linked to later developmental events, coinciding with neurobiological changes during adolescence and early adulthood, and often additionally influenced by environmental stressors that occur at later stages [38, 39]. The consistent strong effect of the ADHD-PGS in both childhood and adulthood challenges the prevailing perception of ADHD as a relatively mild childhood condition that individuals tend to outgrow during development and a condition less likely to require treatment [40, 41]. Our work highlights the importance of research to understand the etiology, and long-term impact of ADHD, along with other psychiatric disorders, which can have far-reaching consequences on various aspects of individuals’ lives, extending beyond those directly affected by the disorders themselves.

In assessing quality of life, we employed a comprehensive approach by integrating self-perceived [5] and objectively quantified impairment in adaptive functioning measures across diverse domains, informants, and scales, and extended beyond the health-related quality of life as often examined in the context of psychiatric disorders [42]. The derived constructs align with individuals’ subjective satisfaction in corresponding life domains. Our findings advocate for an increased emphasis on researching and incorporating the concept of quality of life into clinical practices, thus complementing the conventional focus on reducing psychiatric symptoms as the primary measure of intervention effectiveness.

The utilization of PGSs in this study has allowed for estimation of genetic risk proxies in cohorts without the requirement for specific symptom or trait measures, providing insights into the contribution of different psychiatric disorders to the variables of interest. PGSs offer a cost-effective and efficient means to approximate genetic risk burden and capture the cumulative effects of multiple genetic variants associated with a particular disorder in large general population datasets [18]. By leveraging PGSs, we were able to assess the relative contributions of various disorders to the overall quality of life and its subdomains. The negative relationships we identified between genetic liability to major psychiatric disorders and quality of life related outcomes were consistent across different PGS approaches, despite their small effect sizes. While PGSs offer advantages such as scalability and broad applicability, the common variants captured only reflect a fraction of the total heritability of the disorders and can thus explain a small portion of variance in their primary phenotypes (i.e., diagnostic status) [43]. Joint efforts to increase the sample sizes and ethnic diversity of GWASs, as well as exploiting data for rare and structural genetic variants are essential to provide a more complete individual genetic risk profiles for psychiatric disorders.

Caution should be exercised when interpreting our study due to certain limitations. First, prior research [44] suggested a ‘healthy volunteer selection bias’ in the UK Biobank cohort, where the sample was enriched in wealthier and healthier individuals. This may limit the generalizability of the current results, especially for disorders such as SCZ and ASD, where the debilitating genetic effect might be more pronounced in the samples at the higher end of the liability spectrum. Second, this study only provided snapshots of childhood (around 10 years old) and a part of adulthood (40–70 years old). Longitudinal data are needed to further elucidate how the genetic risks manifest along the trajectory of human development and aging.

In summary, combining newly available GWAS results with genotyped and richly phenotyped cohorts of children and adults, our study highlights the inverse relationship between psychiatric liability and different domains of quality of life. PGSs provided a means to evaluate the contributions of genetic liability for different psychiatric disorders to different aspects of life in the general population.

## Supplementary information

Supplementary Information

Supplementary Tables

## Data Availability

This research has been conducted using data from UK Biobank (http://www.ukbiobank.ac.uk/), under application 23668, and the ABCD Study (https://abcdstudy.org/), under request 11315.

## Code availability

Analysis code is available from the corresponding author on request.
