# Supplementary material for: Genetic liability to major psychiatric disorders contributes to multi-faceted quality of life outcomes in children and adults
Source: Transl Psychiatry. 2025 Jul 7;15:232. doi: 10.1038/s41398-025-03443-y (PMC12234699; doi:10.1038/s41398-025-03443-y)
Supplement: Supplementary file 1 — Supplementary Information [file 41398_2025_3443_MOESM1_ESM.docx]

**Supplementary information**

**Genetic liability to major psychiatric disorders contributes to multi-faceted quality of life outcomes in children and adults**

**eMethods**

**eFigure 1.** Alternative CFA model structures for the UK Biobank and ABCD cohorts.

**eFigure 2.** Variance explained by polygenic scores derived through three approaches in quality of life latent factors for ABCD cohort.

**eFigure 3.** Variance explained by polygenic scores derived through three approaches in quality of life latent factors for UK Biobank cohort.

**eReferences**

Corresponding email:

[yingjie.shi@radboudumc.nl](mailto:yingjie.shi@radboudumc.nl)

**eMethods**

**Genotyping, Quality Control, and Imputation of the target datasets**

Details of the quality control and imputation processes in both cohorts have been described previously ^1,2^. Briefly, UK Biobank participants were genotyped using either the Affymetrix UK BiLEVE Axiom array or the UK Biobank Axiom array^3,4^. A set of stringent quality control (QC) procedures was performed, followed by further imputation using the Haplotype Reference Consortium, UK10K and 1000 Genomes phase 3 reference panels, which resulted in a dataset of 93,095,623 variants.

We further applied sample and variant filtering prior to polygenic score analyses based on the following protocol. We removed individuals with NHS-recorded/self-reported sex and genetic sex mismatch, sample missingness larger than 0.05, and those of non-European descent based on self-report and principal components analysis (PCA) of the genotypes. The principal components (PCs) were provided by the UKBB data analytic team and were used to identify outliers according to PC1 and PC2 after removing ‘non-White’ individuals based on self-report. To account for sample relatedness, we identified related individual pairs with KING kinship coefficients higher than 0.0884 (first- and second-degree relatives) and created family clusters. A greedy algorithm (<https://rdrr.io/cran/ukbtools/man/ukb_gen_samples_to_remove.html>) was applied to trim the connections until only one individual was left within each cluster. Single nucleotide polymorphisms (SNPs) were excluded if they had a Hardy-Weinberg equilibrium (HWE) test P-value < 1e−6, genotype missing rate > 0.05, minor allele frequency (MAF) < 0.005, or imputation quality of INFO < 0.8, resulting a final list of 10,203,392 SNPs.

The ABCD cohort was genotyped using the Affymetrix National Institute on Drug Abuse SmokeScreen Array^5^. Standard pre-imputation QC was conducted following the recommendations of the Ricopili pipeline^6^, after which the resulted variants were imputed to the Trans-Omics for Precision Medicine (TOPMed) panel. The 307,467,480 SNPs were further filtered according to the following criteria: imputation R^2^ > 0.8, MAF > 0.005, HWE p > 1e-6, genotype missing rate < 0.05, yielding 10,537,977 variants. A subset of SNPs with MAF > 0.05 and linkage disequilibrium-pruned with 500kb window size and r^2^ = 0.2 was included to calculate genetic principal components (PCs). Individuals that fall more than three standard deviations beyond the center of the European reference cluster in the 1000 Genome Project^7^ were excluded. We identified unrelated participants, by removing all but one participant per family.

**Polygenic scores calculation**

Polygenic scores were derived and compared using three approaches: 1) classical clumping and thresholding with the p-value that yields the best fit among eight P-value thresholds (P_T_ = 1e-6, 1e-4, 0.001, 0.01, 0.05, 0.1, 0.5, 1) (C+T approach)^8,9^; 2) a principal component approach (PRS-PCA)^10^; and 3) a Bayesian-based continuous shrinkage method (PRS-CS-auto)^11^, as reported in the main text. We held out around one-eighth of the UK Biobank sample as an independent tuning set (N = 37,766) in the C+T approach, where the p threshold that gave the best model fit was selected and evaluated in the test set (N = 269,293). To keep the sample size consistent, the association tests of the scores derived from PRS-PCA and PRS-CS methods were also conducted in the same test set. Given the relatively smaller sample size in the ABCD study (N = 3,909 with complete PGSs and estimated factor scores), we did not further split this cohort and optimized the threshold using the full sample in the C+T approach. For the PRS-PCA approach, the first principal component of polygenic scores (PRS-PC1) derived from *a priori* specified thresholds reweighted the variants for maximum variation. In the PRS-CS approach, a global shrinkage parameter was automatically learned from GWAS summary statistics and imposed on the SNP effect sizes.

**PGS power analyses**

To give an indication of the statistical power of the PGS analyses, we conducted a post-hoc power calculation using the ‘avengeme’ package ^12^ in both cohorts and provided the estimates at different genetic correlation estimates (r_g_ = 0.2, 0.4, 0.6, 0.8, 1.0). The calculation was based on the classical C+T method, which has been shown to be a good approximation ^13^ to the joint modeling of correlated variants and can be used as a rough lower bound for the power estimates using the PRS-CS approach.

**
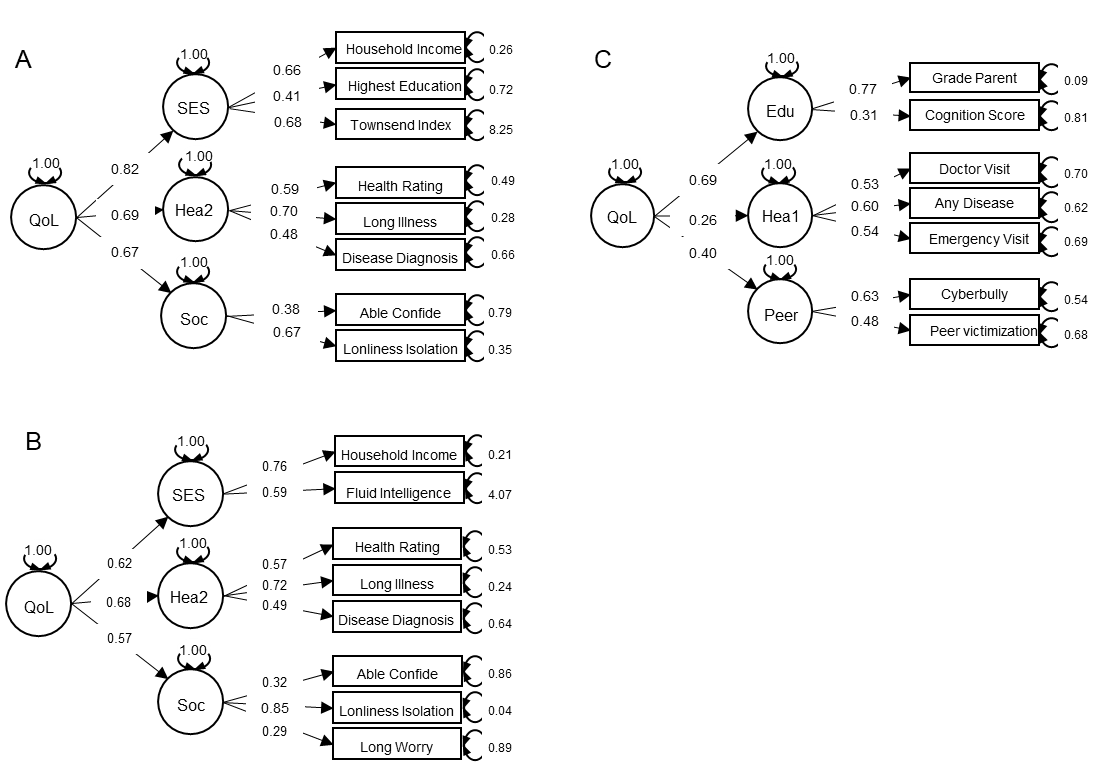
**

**eFigure 1. Alternative CFA model structures for the UK Biobank (A, B) and ABCD (C) cohorts. UK Biobank:** **A)** CFI = 0.969, RMSEA = 0.049, SRMR = 0.048, TLI = 0.948; **B)** CFI = 0.969, RMSEA = 0.042, SRMR = 0.040, TLI = 0.948. **ABCD: C)** CFI = 0.977, RMSEA = 0.031, SRMR = 0.032, TLI = 0.956. QoL – quality of life; SES - socioeconomic status; Hea2 - physical health modeled in UK biobank cohort; Soc – social wellbeing; Edu - educational performance and cognition; Hea1 - physical health modeled in ABCD cohort; Peer - peer experience;.


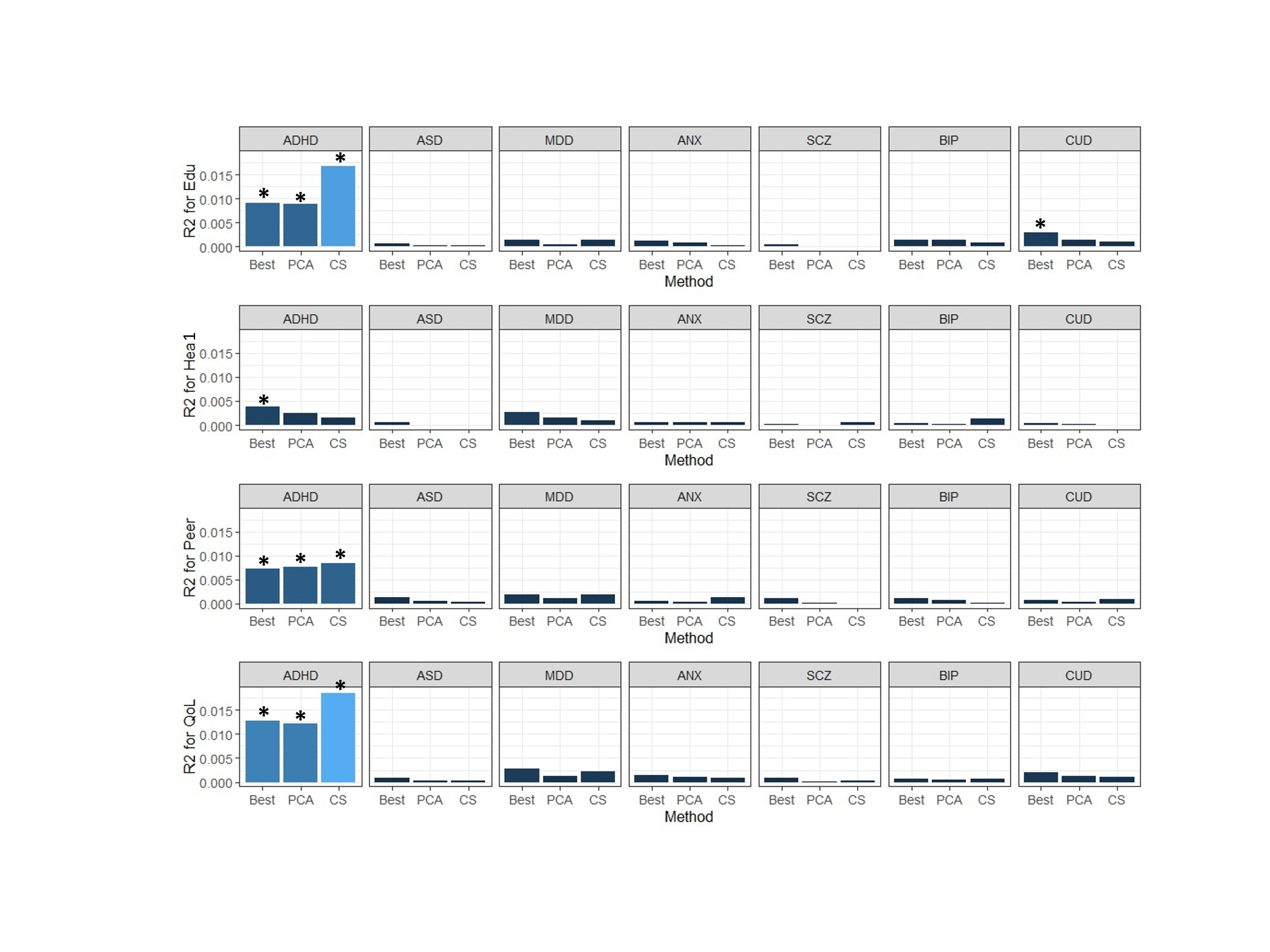


**eFigure 2. Variance explained by polygenic scores derived through three approaches in quality of life latent factors for ABCD cohort.** Edu - educational performance and cognition; Hea1 - physical health; Peer - Peer experience; QoL – general quality of life. Best: clumping and thresholding with the p-value that yields the best fit; PCA: the first principal component of polygenic scores across eight pre-specified thresholds; CS: continuous shrinkage of effect sizes with the global shrinkage parameter automatically learnt from the data. Asterisks indicate significance after multiple test corrections.


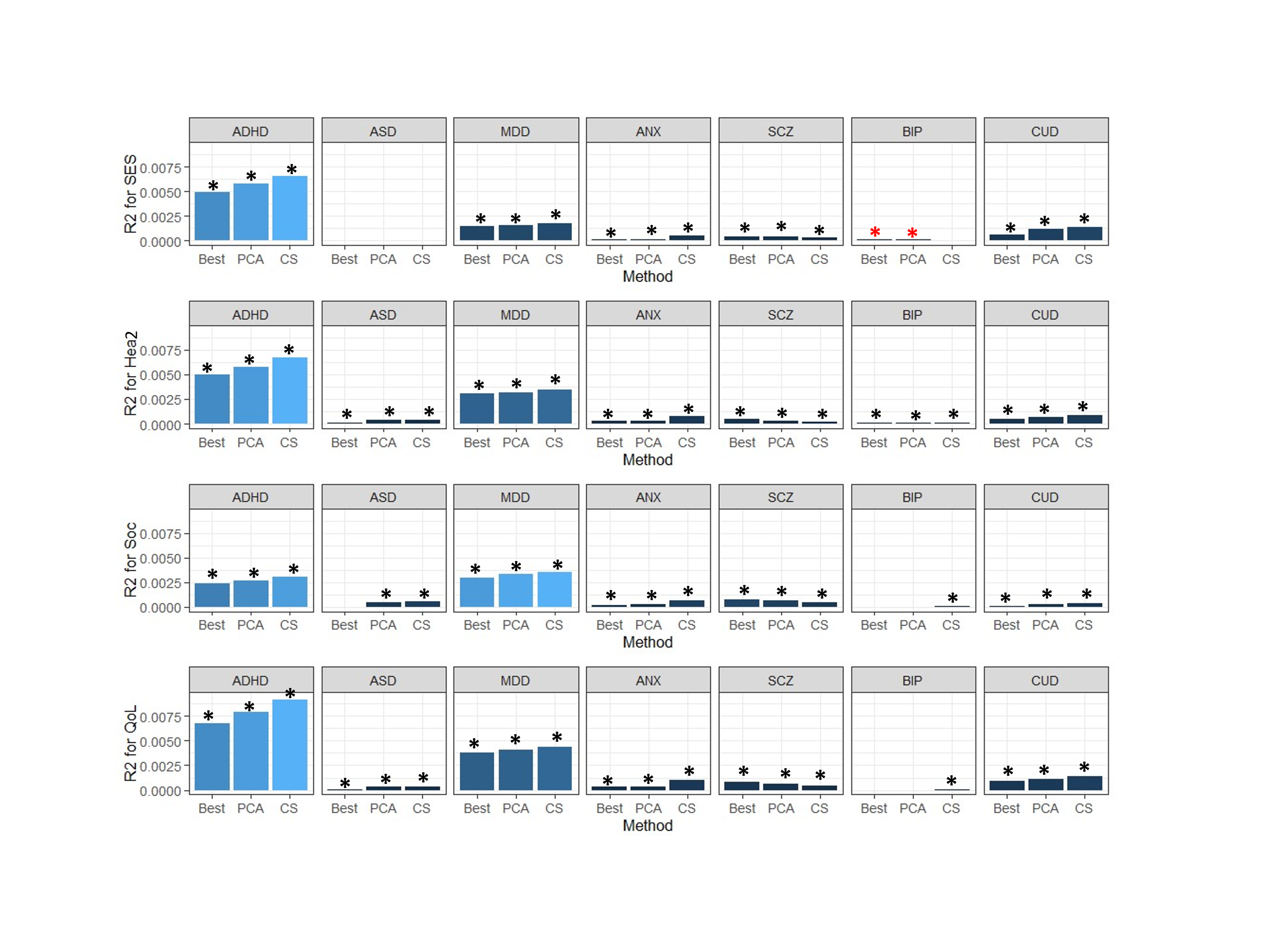


**eFigure 3.** **Variance explained by polygenic scores derived through three approaches in quality of life latent factors for UK Biobank cohort.** SES - social economic status; Hea2 - physical health; Soc - social well-being; QoL – general quality of life. Best: clumping and thresholding with the p-value that yields the best fit; PCA: the first principal component of polygenic scores across eight pre-specified thresholds; CS: continuous shrinkage of effect sizes with the global shrinkage parameter automatically learnt from the data. Asterisks indicate significance after multiple test corrections. Red asterisks indicate significant effect in the opposite direction, i.e., higher PGS, higher quality of life outcome.

**eReferences**

1. Bycroft, C. *et al.* The UK Biobank resource with deep phenotyping and genomic data. *Nature* **562**, 203–209 (2018).

2. Jernigan, T. L., Brown, S. A. & Dowling, G. J. The Adolescent Brain Cognitive Development Study. *J Res Adolesc* **28**, 154–156 (2018).

3. Wain, L. V. *et al.* Novel insights into the genetics of smoking behaviour, lung function, and chronic obstructive pulmonary disease (UK BiLEVE): a genetic association study in UK Biobank. *The Lancet Respiratory Medicine* **3**, 769–781 (2015).

4. Scientific, T. F. UKB_WCSGAX: UK Biobank 500K Samples Genotyping Data Generation by the Affymetrix Research Services Laboratory.

5. Baurley, J. W., Edlund, C. K., Pardamean, C. I., Conti, D. V. & Bergen, A. W. Smokescreen: a targeted genotyping array for addiction research. *BMC Genomics* **17**, 145 (2016).

6. Lam, M. *et al.* RICOPILI: Rapid Imputation for COnsortias PIpeLIne. *Bioinformatics* **36**, 930–933 (2020).

7. Fairley, S., Lowy-Gallego, E., Perry, E. & Flicek, P. The International Genome Sample Resource (IGSR) collection of open human genomic variation resources. *Nucleic Acids Research* **48**, D941–D947 (2020).

8. Wray, N. R. *et al.* Research Review: Polygenic methods and their application to psychiatric traits. *Journal of Child Psychology and Psychiatry* **55**, 1068–1087 (2014).

9. Choi, S. W. & O’Reilly, P. F. PRSice-2: Polygenic Risk Score software for biobank-scale data. *GigaScience* **8**, giz082 (2019).

10. Coombes, B. J., Ploner, A., Bergen, S. E. & Biernacka, J. M. A principal component approach to improve association testing with polygenic risk scores. *Genetic Epidemiology* **44**, 676–686 (2020).

11. Ge, T., Chen, C.-Y., Ni, Y., Feng, Y.-C. A. & Smoller, J. W. Polygenic prediction via Bayesian regression and continuous shrinkage priors. *Nat Commun* **10**, 1776 (2019).

12. Dudbridge, F. Power and Predictive Accuracy of Polygenic Risk Scores. *PLOS Genetics* **9**, e1003348 (2013).

13. Dudbridge, F. & Newcombe, P. J. Accuracy of Gene Scores when Pruning Markers by Linkage Disequilibrium. *Hum Hered* **80**, 178–186 (2015).
