# Supplementary material for: Genetic liability to major psychiatric disorders contributes to multi-faceted quality of life outcomes in children and adults
Source: Transl Psychiatry. 2025 Jul 7;15:232. doi: 10.1038/s41398-025-03443-y (PMC12234699; doi:10.1038/s41398-025-03443-y)
Supplement: Supplementary file 2 — Supplementary Tables [file 41398_2025_3443_MOESM2_ESM.pdf]

Supplementary Tables

**eTable 1**	**Overview of indicators from the ABCD study included in the primary and/or alternative model.**
**eTable 2**	**Overview of indicators from the UK Biobank study included in the primary and/or alternative model.**
**eTable 3**	**Model parameters of the primary model (ABCD).**
**eTable 4**	**Model parameters of the primary model (UK Biobank).**
**eTable 5**	**Spearman's correlation between QoL latent domains and self-reported satisfaction towards diverse aspects of life.**
**eTable 6**	**Test statistics of associations between polygenic scores and latent factors in two cohorts.**
**eTable 7**	**PGS power calculation with different degrees of genetic correlation, using the C+T method as an approximation in the ABCD cohort.**
**eTable 8**	**PGS power calculation with different genetic correlation estimates, using the C+T method as an approximation in the UK Biobank cohort.**
**eTable 9**	**Variance explained by regression models consisting of all 7 PGSs, and each single PGS, proportional to the SNP heritability estimated from the GWA analyses in UK Biobank**
**eTable 10**	**Genetic correlations based on LD score regression between psychiatric disorders and latent factors in UK Biobank**

eTable 1. Overview of indicators from the ABCD study included in the primary and/or alternative model.

Latent factor	Questionnaire/ task	Description	Abbrev.	N	Timepoint	Type
Educational performance and cognition (Edu)	Parent School Attendance and Grades	What grades did your child receive in school last year? (sag_grade_type)	Grade.Parent	4,104	2_year_follow_up	Ordinal – 12, treated as continuous
	Youth School Attendance and Grades	What grades did you receive in school last year? (sag_grades_last_yr)	Grade.Child	4,135	2_year_follow_up	Ordinal – 12, treated as continuous
	Youth NIH TB Summary Scores	Cognition Total Composite Score Uncorrected Standard Score (nihtbx_totalcomp_uncorrected)	Cognition.Score	4,625	Baseline_1_year	Continuous
Physical Health (Hea1)	Parent Medical History Questionnaire	Ever seen doctor before past year (excl. check-ups) (medhx_1b)	Doctor.Visit	4,625	Baseline_1_year	Binary
		Seen doctor for any (severe) diseases; derived based on medhx_2a to medhx_2q, excluding medhx_2b and medhx_2n	Any.Disease	4,644		Binary
		Emergency room visits before past year. Derived by combining medhx_5a and medhx_5b	Emergency.Visit	4,602		Ordinal - 5
		Ever been in the hospital overnight or longer (medhx_8a)	Hospital.Overnight	4,630		Binary
Peer Experience (Peer)	Cyber bully	Have you ever been cyberbullied, where someone was trying on purpose to harm you or be mean to you online, in texts, or group texts, or on social media (like Instagram or Snapchat)? (cybb_phenx_harm)	Cyberbully	4,205	2_year_follow_up	Binary
	Peer experience questionnaire	Whether the youth has either experienced overt, relational or reputational victimization from peers	Peer.Victimization	4,216	2_year_follow_up	Continuous

eTable 2. Overview of indicators from the UK Biobank study included in the primary and/or alternative model.

Latent factor	UKB Index	Description	Abbrev.	N	Type
**Socioeconomic Status (SES)**	738	Average total household income before tax	Household.Income	497,375	Ordinal-5
	6138	ISCED coded highest qualifications	Highest.Education	498,734	Ordinal-5
	189	Townsend deprivation index at recruitment	Townsend.Index	501,790	Continuous
	20016	Fluid intelligence score	Fluid.Intelligence	229,668	Continuous
**Physical Health (Hea2)**	2178	Overall health self-rating	Health.Rating	501,513	Ordinal-4
	2188	Long-standing illness, disability or infirmity	Long.Illness	501,513	Binary-2
	6150<br>6152<br>2443<br>2453<br>2473	Diagnosed with any serious medical conditions	Disease.Diagnosis	496,999	Binary -2
**Social Wellbeing (Soc)**	2110	Frequency of being able to confide	Able.Confide	501,519	Ordinal-6
	2020	Often feel loneliness or in isolation	Lonliness.Isolation	501,524	Binary-2
	2000	Worry too long after embarrassment	Long.Worry	501,523	Binary -2

eTable 3. Model parameters of the primary model (ABCD study).

Latent Variables	Estimate	Std.Err	z-value	P(>|z|)	Std.lv	Std.all
Edu	=~					
Grade.Parent	0.74	0.067	11.06	0	0.897	0.909
Grade.Child	0.532	0.047	11.376	0	0.645	0.655
Cognition.Scor	0.315	0.029	10.816	0	0.382	0.392
Hea1	=~					
Doctor.Visit	0.475	0.032	15.013	0	0.495	0.495
Any.Disease	0.526	0.028	18.66	0	0.548	0.548
Emergency.Vist	0.626	0.029	21.61	0	0.652	0.652
Hosptl.Ovrnght	0.534	0.03	17.524	0	0.557	0.557
Peer	=~					
Cyberbully	0.592	0.06	9.914	0	0.644	0.644
Peer.Victimztn	0.501	0.059	8.529	0	0.544	0.56
QoL	=~					
Edu	0.685	0.182	3.756	0	0.565	0.565
Hea1	0.295	0.063	4.653	0	0.283	0.283
Peer	0.427	0.103	4.156	0	0.392	0.392
**Variances**	**Estimate**	**Std.Err**	**z-value**	**P(>|z|)**	**Std.lv**	**Std.all**
.Edu	1	0.68	0.68			
.Hea1	1	0.92	0.92			
.Peer	1	0.846	0.846			
QoL	1	1	1			
.Grade.Parent	0.168	0.034	4.946	0	0.168	0.173
.Grade.Child	0.554	0.021	26.863	0	0.554	0.571
.Cognition.Scor	0.803	0.018	45.895	0	0.803	0.846
.Doctor.Visit	0.755	0.755	0.755			
.Any.Disease	0.699	0.699	0.699			
.Emergency.Vist	0.574	0.574	0.574			
.Hosptl.Ovrnght	0.69	0.69	0.69			
.Cyberbully	0.585	0.585	0.585			
.Peer.Victimztn	0.649	0.06	10.887	0	0.649	0.687

eTable 4. Model parameters of the primary model (UK Biobank).

Latent Variables	Estimate	Std.Err	z-value	P(>|z|)	Std.lv	Std.all
SES	=~					
Household.Incm	0.755	0.006	130.358	0	0.899	0.899
Highest.Eductn	0.445	0.002	188.082	0	0.529	0.529
Hea2	=~					
Hea2lth.Rating	0.561	0.003	187.296	0	0.708	0.708
Long.Illness	0.674	0.003	196.651	0	0.85	0.85
Disease.Dignss	0.464	0.003	173.373	0	0.586	0.586
Soc	=~					
Able.Confide	0.363	0.003	133.512	0	0.414	0.414
Lonlinss.Isltn	0.817	0.006	131.406	0	0.934	0.934
Long.Worry	0.285	0.003	102.802	0	0.325	0.325
QoL	=~					
SES	0.647	0.008	83.869	0	0.543	0.543
Hea2	0.769	0.009	85.618	0	0.61	0.61
Soc	0.554	0.006	85.802	0	0.485	0.485
**Variances**	**Estimate**	**Std.Err**	**z-value**			
.Household.Incm	0.192	0.192	0.192			
.Highest.Eductn	0.72	0.72	0.72			
.Hea2lth.Rating	0.499	0.499	0.499			
.Long.Illness	0.278	0.278	0.278			
.Disease.Dignss	0.657	0.657	0.657			
.Able.Confide	0.828	0.828	0.828			
.Lonlinss.Isltn	0.127	0.127	0.127			
.Long.Worry	0.894	0.894	0.894			
.SES	1	0.705	0.705			
.Hea2	1	0.628	0.628			
.Soc	1	0.765	0.765			
QoL	1	1	1			

eTable 5. Spearman's correlation between QoL latent domains and self-reported satisfaction towards diverse aspects of life.

Coefficient	General Happiness	Job Satisfaction	Finance Satisfaction	Health Satisfaction	Family Satisfaction	Friends Satisfaction
**N**	80,255	58,632	80,343	80,342	79,907	79,851
**SES**	0.1	0.04	0.25	0.17	0.06	0
**Hea2**	0.21	0.12	0.22	0.58	0.15	0.13
**Soc**	0.41	0.21	0.27	0.32	0.32	0.28

Note . All correlations, except SES-Friends Satisfaction, were significant with $p < 0.001$.

eTable 6. Test statistics of associations between polygenic scores and latent factors in two cohorts.

		std. est.	SE	t	p	R2	std. est.	SE	t	p	R2
		Edu					Hea1				
ABCD	ADHD	-0.133	0.016	-8.292	1.53E-16	1.67E-02	-0.040	0.016	-2.461	1.39E-02	1.51E-03
	ASD	-0.013	0.016	-0.821	4.12E-01	1.66E-04	-0.008	0.016	-0.517	6.05E-01	6.65E-05
	MDD	-0.038	0.016	-2.347	1.90E-02	1.36E-03	-0.031	0.016	-1.901	5.73E-02	8.99E-04
	ANX	-0.016	0.016	-0.992	3.21E-01	2.43E-04	-0.023	0.016	-1.453	1.46E-01	5.25E-04
	SCZ	0.012	0.016	0.756	4.50E-01	1.41E-04	0.026	0.016	1.620	1.05E-01	6.53E-04
	BIP	0.029	0.016	1.798	7.22E-02	7.98E-04	0.037	0.016	2.289	2.21E-02	1.30E-03
	CUD	-0.032	0.016	-1.960	5.00E-02	9.48E-04	0.001	0.016	0.060	9.52E-01	9.01E-07
		Peer					QoL				
	ADHD	-0.094	0.016	-5.786	7.81E-09	8.43E-03	-0.140	0.016	-8.743	3.37E-18	1.85E-02
	ASD	-0.017	0.016	-1.015	3.10E-01	2.62E-04	-0.017	0.016	-1.054	2.92E-01	2.74E-04
	MDD	-0.044	0.016	-2.693	7.11E-03	1.84E-03	-0.049	0.016	-3.047	2.32E-03	2.29E-03
	ANX	-0.038	0.016	-2.325	2.01E-02	1.37E-03	-0.029	0.016	-1.782	7.48E-02	7.83E-04
	SCZ	0.006	0.016	0.378	7.05E-01	3.63E-05	0.017	0.016	1.059	2.90E-01	2.77E-04
	BIP	-0.016	0.016	-0.953	3.41E-01	2.31E-04	0.026	0.016	1.625	1.04E-01	6.52E-04
	CUD	-0.031	0.016	-1.876	6.08E-02	8.93E-04	-0.033	0.016	-2.071	3.85E-02	1.06E-03

		std. est.	SE	t	p	R2	std. est.	SE	t	p	R2
		Edu					Hea2				
UK Biobank	ADHD	-0.081	0.002	-46.508	<2,23E-308	6.56E-03	-0.083	0.002	-43.736	<2,23E-308	6.77E-03
	ASD	-0.002	0.002	-0.900	3.68E-01	2.48E-06	-0.019	0.002	-9.919	3.47E-23	3.50E-04
	MDD	-0.042	0.002	-23.809	3.63E-125	1.73E-03	-0.059	0.002	-31.034	4.48E-211	3.42E-03
	ANX	-0.022	0.002	-12.624	1.60E-36	4.87E-04	-0.028	0.002	-15.035	4.57E-51	8.05E-04
	SCZ	-0.017	0.002	-9.687	3.46E-22	2.87E-04	-0.014	0.002	-7.209	5.66E-13	1.85E-04
	BIP	0.003	0.002	1.622	1.05E-01	8.04E-06	-0.012	0.002	-6.225	4.83E-10	1.38E-04
	CUD	-0.037	0.002	-21.173	2.05E-99	1.37E-03	-0.030	0.002	-15.898	6.93E-57	9.00E-04
		Soc					QoL				
	ADHD	-0.056	0.002	-29.106	5.91E-186	3.09E-03	-0.096	0.002	-52.500	<2,23E-308	9.13E-03
	ASD	-0.024	0.002	-12.604	2.06E-36	5.81E-04	-0.017	0.002	-9.436	3.88E-21	2.98E-04
	MDD	-0.060	0.002	-31.230	9.96E-214	3.56E-03	-0.066	0.002	-36.313	4.97E-288	4.39E-03
	ANX	-0.025	0.002	-13.138	2.06E-39	6.31E-04	-0.032	0.002	-17.471	2.58E-68	1.02E-03
	SCZ	-0.021	0.002	-10.721	8.20E-27	4.20E-04	-0.021	0.002	-11.261	2.07E-29	4.24E-04
	BIP	-0.011	0.002	-5.488	4.07E-08	1.10E-04	-0.008	0.002	-4.256	2.08E-05	6.06E-05
	CUD	-0.019	0.002	-9.825	8.83E-23	3.53E-04	-0.038	0.002	-20.697	4.36E-95	1.43E-03

Note . std. est. - standard estimates; SE - standard error; R2 - variance explained after accounting for covariates.

eTable 7. PGS power calculation with different degrees of genetic correlation, using the C+T method as an approximation in the ABCD cohort.

Target phenotype	Base phenotype	n_discovery	n_target	n_snps	vg1	vg2	Population prevalence	Sample prevalence	rg=0,2	rg=0,4	rg=0,6	rg=0,8	rg=1,0
**Edu**	**ADHD**	225534	4645	207499	0.140	0.100	0.050	0.172	0.451	0.957	1.000	1.000	1.000
**Edu**	**ASD**	55421	4645	333426	0.120	0.100	0.012	0.414	0.122	0.343	0.645	0.875	0.973
**Edu**	**MDD**	173005	4645	155422	0.090	0.100	0.150	0.346	0.304	0.825	0.992	1.000	1.000
**Edu**	**ANX**	114091	4645	132391	0.260	0.100	0.160	0.280	0.648	0.997	1.000	1.000	1.000
**Edu**	**SCZ**	130644	4645	150513	0.240	0.100	0.010	0.409	0.606	0.994	1.000	1.000	1.000
**Edu**	**BIP**	413466	4645	157366	0.186	0.100	0.010	0.101	0.566	0.989	1.000	1.000	1.000
**Edu**	**CUD**	384032	4645	259463	0.100	0.100	0.050	0.054	0.126	0.360	0.670	0.893	0.980
**Heal**	**ADHD**	225534	4645	207499	0.140	0.070	0.050	0.172	0.336	0.867	0.996	1.000	1.000
**Heal**	**ASD**	55421	4645	333426	0.120	0.070	0.012	0.414	0.100	0.255	0.496	0.740	0.902
**Heal**	**MDD**	173005	4645	155422	0.090	0.070	0.150	0.346	0.227	0.678	0.953	0.998	1.000
**Heal**	**ANX**	114091	4645	132391	0.260	0.070	0.160	0.280	0.499	0.975	1.000	1.000	1.000
**Heal**	**SCZ**	130644	4645	150513	0.240	0.070	0.010	0.409	0.462	0.962	1.000	1.000	1.000
**Heal**	**BIP**	413466	4645	157366	0.186	0.070	0.010	0.101	0.429	0.946	1.000	1.000	1.000
**Heal**	**CUD**	384032	4645	259463	0.100	0.070	0.050	0.054	0.103	0.268	0.519	0.764	0.918
**Peer**	**ADHD**	225534	4645	207499	0.140	0.050	0.050	0.172	0.255	0.738	0.974	0.999	1.000
**Peer**	**ASD**	55421	4645	333426	0.120	0.050	0.012	0.414	0.085	0.196	0.378	0.594	0.785
**Peer**	**MDD**	173005	4645	155422	0.090	0.050	0.150	0.346	0.176	0.534	0.867	0.984	0.999
**Peer**	**ANX**	114091	4645	132391	0.260	0.050	0.160	0.280	0.380	0.912	0.999	1.000	1.000
**Peer**	**SCZ**	130644	4645	150513	0.240	0.050	0.010	0.409	0.351	0.884	0.997	1.000	1.000
**Peer**	**BIP**	413466	4645	157366	0.186	0.050	0.010	0.101	0.324	0.853	0.995	1.000	1.000
**Peer**	**CUD**	384032	4645	259463	0.100	0.050	0.050	0.054	0.087	0.205	0.396	0.619	0.808
**QoL**	**ADHD**	225534	4645	207499	0.140	0.080	0.050	0.172	0.375	0.908	0.999	1.000	1.000
**QoL**	**ASD**	55421	4645	333426	0.120	0.080	0.012	0.414	0.107	0.285	0.550	0.795	0.936
**QoL**	**MDD**	173005	4645	155422	0.090	0.080	0.150	0.346	0.253	0.735	0.973	0.999	1.000
**QoL**	**ANX**	114091	4645	132391	0.260	0.080	0.160	0.280	0.553	0.987	1.000	1.000	1.000
**QoL**	**SCZ**	130644	4645	150513	0.240	0.080	0.010	0.409	0.514	0.979	1.000	1.000	1.000
**QoL**	**BIP**	413466	4645	157366	0.186	0.080	0.010	0.101	0.477	0.968	1.000	1.000	1.000
**QoL**	**CUD**	384032	4645	259463	0.100	0.080	0.050	0.054	0.110	0.299	0.574	0.817	0.948

Note . nsnp - number of independent markers (after LD clumping) in the polygenic score; vg1 – SNP heritability of trait in base GWAS; vg2 – SNP heritability of trait in target sample. 5% of SNPs were assumed to have an effect on the base trait, and the power estimates were maximized across the specified p thresholds. We estimated the SNP heritability of the latent factors based on previous study of similar traits. The population prevalences were obtained from the original GWAS publication. The resulted estimation should indicate the lower bound of the statistical power of the PGS analyses.

eTable 8. PGS power calculation with different genetic correlation estimates, using the C+T method as an approximation in the UK Biobank cohort.

Target phenotype	Base phenotype	n_discovery	n_target	n_snps	vg1	vg2	Population prevalence	Sample prevalence	rg=0,2	rg=0,4/0,6/0,8/1
**SES**	**ADHD**	225534	269293	220786	0.140	0.113	0.050	0.172	1.000	1.000
**SES**	**ASD**	55421	269293	377126	0.120	0.113	0.012	0.414	1.000	1.000
**SES**	**MDD**	143265	269293	148809	0.090	0.113	0.150	0.318	1.000	1.000
**SES**	**ANX**	21761	269293	155399	0.260	0.113	0.160	0.322	1.000	1.000
**SES**	**SCZ**	130644	269293	159948	0.240	0.113	0.010	0.409	1.000	1.000
**SES**	**BIP**	353899	269293	159321	0.186	0.113	0.010	0.114	1.000	1.000
**SES**	**CUD**	384032	269293	282695	0.100	0.113	0.050	0.054	1.000	1.000
**Hea2**	**ADHD**	225534	269293	220786	0.140	0.094	0.050	0.172	1.000	1.000
**Hea2**	**ASD**	55421	269293	377126	0.120	0.094	0.012	0.414	1.000	1.000
**Hea2**	**MDD**	143265	269293	148809	0.090	0.094	0.150	0.318	1.000	1.000
**Hea2**	**ANX**	21761	269293	155399	0.260	0.094	0.160	0.322	1.000	1.000
**Hea2**	**SCZ**	130644	269293	159948	0.240	0.094	0.010	0.409	1.000	1.000
**Hea2**	**BIP**	353899	269293	159321	0.186	0.094	0.010	0.114	1.000	1.000
**Hea2**	**CUD**	384032	269293	282695	0.100	0.094	0.050	0.054	1.000	1.000
**Soc**	**ADHD**	225534	269293	220786	0.140	0.063	0.050	0.172	1.000	1.000
**Soc**	**ASD**	55421	269293	377126	0.120	0.063	0.012	0.414	0.993	1.000
**Soc**	**MDD**	143265	269293	148809	0.090	0.063	0.150	0.318	1.000	1.000
**Soc**	**ANX**	21761	269293	155399	0.260	0.063	0.160	0.322	1.000	1.000
**Soc**	**SCZ**	130644	269293	159948	0.240	0.063	0.010	0.409	1.000	1.000
**Soc**	**BIP**	353899	269293	159321	0.186	0.063	0.010	0.114	1.000	1.000
**Soc**	**CUD**	384032	269293	282695	0.100	0.063	0.050	0.054	0.996	1.000
**QoL**	**ADHD**	225534	269293	220786	0.140	0.115	0.050	0.172	1.000	1.000
**QoL**	**ASD**	55421	269293	377126	0.120	0.115	0.012	0.414	1.000	1.000
**QoL**	**MDD**	143265	269293	148809	0.090	0.115	0.150	0.318	1.000	1.000
**QoL**	**ANX**	21761	269293	155399	0.260	0.115	0.160	0.322	1.000	1.000
**QoL**	**SCZ**	130644	269293	159948	0.240	0.115	0.010	0.409	1.000	1.000
**QoL**	**BIP**	353899	269293	159321	0.186	0.115	0.010	0.114	1.000	1.000
**QoL**	**CUD**	384032	269293	282695	0.100	0.115	0.050	0.054	1.000	1.000

Note . nsnp - number of independent markers (after LD clumping) in the polygenic score; vg1 – SNP heritability of trait in base GWAS; vg2 – SNP heritability of trait in target sample; 5% of SNPs were assumed to have an effect on the base trait, and the power estimates were maximized across the specified p thresholds. The estimates of SNP heritability and population prevalence of the disorders were obtained from the original GWAS publication. The resulted estimation should indicate the lower bound of the statistical power of the PGS analyses.

eTable 9. Variance explained by regression models consisting of all 7 PGSs, and each single PGS, proportional to the SNP heritability estimated from the GWA analyses in UK Biobank.

	SES	Hea2	Soc	QoL
**h^2_{SNP}**	0.113	0.094	0.063	0.115
**h2 - SE**	0.005	0.004	0.003	0.005
**All PGS**	0.097	0.104	0.097	0.122
**ADHD**	0.058	0.072	0.049	0.079
**ASD**	0.000	0.004	0.013	0.003
**MDD**	0.015	0.036	0.079	0.038
**ANX**	0.004	0.009	0.014	0.009
**SCZ**	0.003	0.002	0.009	0.004
**BIP**	0.000	0.001	0.002	0.001
**CUD**	0.012	0.010	0.008	0.012

eTable 10. Genetic correlations based on LD score regression between psychiatric disorders and latent factors in UK Biobank.

p1	p2	rg	SE	Z	p	h2_liab	h2_liab_se	h2_int	h2_int_se	gcov_int	gcov_int_se
SES	ADHD	-0.562	0.022	-25.691	1.47E-145	0.143	0.007	1.027	0.010	-0.009	0.007
	ASD	0.005	0.038	0.126	9.00E-01	0.116	0.010	1.009	0.010	-0.009	0.008
	MDD	-0.373	0.033	-11.370	5.87E-30	0.100	0.007	0.995	0.010	-0.007	0.007
	ANX	-0.485	0.110	-4.423	9.73E-06	0.101	0.040	1.004	0.007	0.003	0.006
	SCZ	-0.121	0.022	-5.583	2.36E-08	0.210	0.008	1.073	0.016	-0.002	0.008
	BIP	0.024	0.025	0.951	3.42E-01	0.113	0.005	1.026	0.012	0.002	0.008
	CUD	-0.400	0.038	-10.480	1.07E-25	0.060	0.006	0.990	0.007	-0.003	0.006
Hea2	ADHD	-0.589	0.023	-25.747	3.45E-146	0.143	0.007	1.027	0.010	-0.004	0.007
	ASD	-0.176	0.041	-4.325	1.52E-05	0.116	0.010	1.009	0.010	-0.007	0.008
	MDD	-0.576	0.034	-17.069	2.55E-65	0.100	0.007	0.995	0.010	-0.001	0.007
	ANX	-0.643	0.142	-4.522	6.11E-06	0.101	0.040	1.004	0.007	0.001	0.006
	SCZ	-0.131	0.023	-5.685	1.31E-08	0.210	0.008	1.073	0.016	-0.008	0.008
	BIP	-0.133	0.024	-5.641	1.69E-08	0.113	0.005	1.026	0.012	0.005	0.007
	CUD	-0.319	0.040	-8.075	6.77E-16	0.060	0.006	0.990	0.007	-0.008	0.006
Soc	ADHD	-0.478	0.027	-17.436	4.41E-68	0.143	0.007	1.027	0.010	-0.004	0.006
	ASD	-0.298	0.044	-6.817	9.29E-12	0.116	0.010	1.009	0.010	0.005	0.007
	MDD	-0.688	0.035	-19.558	3.54E-85	0.100	0.007	0.995	0.010	-0.001	0.007
	ANX	-0.682	0.151	-4.529	5.92E-06	0.101	0.040	1.004	0.007	0.002	0.006
	SCZ	-0.170	0.027	-6.309	2.81E-10	0.210	0.008	1.073	0.016	-0.012	0.008
	BIP	-0.088	0.029	-3.053	2.27E-03	0.113	0.005	1.026	0.012	-0.007	0.007
	CUD	-0.201	0.046	-4.350	1.36E-05	0.060	0.006	0.990	0.007	-0.013	0.006
QoL	ADHD	-0.633	0.020	-32.460	3.97E-231	0.143	0.007	1.027	0.010	-0.007	0.007
	ASD	-0.148	0.036	-4.074	4.62E-05	0.116	0.010	1.009	0.010	-0.006	0.008
	MDD	-0.589	0.031	-18.902	1.10E-79	0.100	0.007	0.995	0.010	-0.003	0.008
	ANX	-0.671	0.139	-4.813	1.48E-06	0.101	0.040	1.004	0.007	0.002	0.006
	SCZ	-0.152	0.022	-6.989	2.77E-12	0.210	0.008	1.073	0.016	-0.009	0.008
	BIP	-0.073	0.023	-3.176	1.49E-03	0.113	0.005	1.026	0.012	0.001	0.007
	CUD	-0.370	0.037	-9.936	2.91E-23	0.060	0.006	0.990	0.007	-0.009	0.006
